# Super-resolution microscopy unveils the nanoscale organization and self-limiting clustering of CD47 in human erythrocytes

**DOI:** 10.1093/jmcb/mjae041

**Published:** 2024-10-04

**Authors:** Jianyu Yang, Fulin Xing, Fen Hu, Mengdi Hou, Hao Dong, Jiayu Cheng, Wan Li, Rui Yan, Jingjun Xu, Ke Xu, Leiting Pan

**Affiliations:** The Key Laboratory of Weak-Light Nonlinear Photonics of Education Ministry, School of Physics and TEDA Institute of Applied Physics, Nankai University, Tianjin 300071, China; The Key Laboratory of Weak-Light Nonlinear Photonics of Education Ministry, School of Physics and TEDA Institute of Applied Physics, Nankai University, Tianjin 300071, China; The Key Laboratory of Weak-Light Nonlinear Photonics of Education Ministry, School of Physics and TEDA Institute of Applied Physics, Nankai University, Tianjin 300071, China; The Key Laboratory of Weak-Light Nonlinear Photonics of Education Ministry, School of Physics and TEDA Institute of Applied Physics, Nankai University, Tianjin 300071, China; The Key Laboratory of Weak-Light Nonlinear Photonics of Education Ministry, School of Physics and TEDA Institute of Applied Physics, Nankai University, Tianjin 300071, China; The Key Laboratory of Weak-Light Nonlinear Photonics of Education Ministry, School of Physics and TEDA Institute of Applied Physics, Nankai University, Tianjin 300071, China; Department of Chemistry, University of California, Berkeley, CA 94720, USA; Department of Chemistry, University of California, Berkeley, CA 94720, USA; The Key Laboratory of Weak-Light Nonlinear Photonics of Education Ministry, School of Physics and TEDA Institute of Applied Physics, Nankai University, Tianjin 300071, China; Shenzhen Research Institute of Nankai University, Shenzhen 518083, China; Department of Chemistry, University of California, Berkeley, CA 94720, USA; The Key Laboratory of Weak-Light Nonlinear Photonics of Education Ministry, School of Physics and TEDA Institute of Applied Physics, Nankai University, Tianjin 300071, China; Shenzhen Research Institute of Nankai University, Shenzhen 518083, China; State Key Laboratory of Medicinal Chemical Biology, Frontiers Science Center for Cell Responses, College of Life Sciences, Nankai University, Tianjin 300071, China

**Keywords:** super-resolution microscopy, human erythrocytes, CD47, self-limiting clustering

## Abstract

The transmembrane protein CD47, an innate immune checkpoint protein, plays a pivotal role in preventing healthy erythrocytes from immune clearance. Our study utilized stochastic optical reconstruction microscopy (STORM) and single-molecule analysis to investigate the distribution of CD47 on the human erythrocyte membrane. Contrary to previous findings in mouse erythrocytes, we discovered that CD47 exists in randomly distributed monomers rather than in clusters across the human erythrocyte membrane. Using secondary antibody-induced crosslinking, we found that CD47 aggregates into stable clusters within minutes. By comparing these STORM results with those of the fully mobile protein CD59 and the cytoskeleton-bound membrane protein glycophorin C under similar conditions, as well as devising two-color STORM co-labeling and co-clustering experiments, we further quantitatively revealed an intermediate, self-limiting clustering behavior of CD47, elucidating its fractional (∼14%) attachment to the cytoskeleton. Moreover, we report reductions in both the amount of CD47 and its clustering capability in aged erythrocytes, providing new insight into erythrocyte senescence. Together, the combination of STORM and secondary antibody-based crosslinking unveils the unique self-limiting clustering behavior of CD47 due to its fractional cytoskeleton attachment.

## Introduction

The transmembrane protein CD47, also known as integrin-associated protein, plays a crucial role as a ‘don't eat me’ signal on cell surfaces, helping cells evade elimination by the immune system (Russ et al., 2018; Veillette and Chen, 2018; Eladl et al., 2020; Logtenberg et al., 2020; Majeti et al., 2022). Initially identified as a marker of ovarian cancer, CD47 was later found to be ubiquitous in different mammalian cell types, leading to a paradigm shift in cancer immunotherapy (Liu et al., 2023a, 2023b). In particular, a high level of CD47 presents at the plasma membrane of red blood cells (erythrocytes), where it partly associates with the spectrin–actin-based membrane cytoskeleton through Band 3 and Protein 4.2 (Bruce et al., 2002; Dahl et al., 2004; Lux, 2016). Notably, upon injection into normal mice, CD47-null erythrocytes are rapidly cleared, which is in contrast to wild-type erythrocytes expressing CD47 (Oldenborg et al., 2000). Meanwhile, although conflicting results have been reported regarding the efficacy of antibody-mediated CD47 blockade in suppressing solid tumors, consistent observations of anemia in these experiments suggest the pivotal role of CD47 in preventing the clearance of healthy erythrocytes *in vivo* (Willingham et al., 2012; Horrigan, 2017).

The organization of membrane proteins has attracted substantial research attention due to both the diverse clustering and motional states and the resultant fundamental biological functions (Garcia-Parajo et al., 2014; Van Deventer et al., 2021). Recent advances in super-resolution fluorescence microscopy have revolutionized our understanding of the cellular structure (Pan et al., 2018; Sigal et al., 2018; Bond et al., 2022; Yan et al., 2022) and function of membrane proteins at the nanoscale (Zhang et al., 2019; Rochussen et al., 2023). In particular, stochastic optical reconstruction microscopy (STORM) has shown that T-cell antigen receptors are randomly distributed on the plasma membrane of resting T cells but become clustered upon activation (Rossboth et al., 2018). Signal regulatory protein α (SIRPα), the receptor of CD47, is organized in discrete nanoclusters on the membrane of human macrophages (Lopes et al., 2017). In mouse erythrocytes, CD47 resides as clusters (Wang et al., 2020). Previous immunofluorescence studies in human erythrocytes (Dahl et al., 2003; Subramanian et al., 2006) and cultured cell lines (Lv et al., 2015) have also suggested patchy, domain-like staining at the micrometer and submicrometer scales. However, the actual spatial distribution pattern of CD47 and its potential clustering capability in the human erythrocyte membrane, as suggested by earlier experiments based on fluorescence-imaged microdeformation (FIMD) (Discher et al., 1994; Dahl et al., 2003), remain elusive.

In the present work, we used STORM to resolve the distribution of CD47 at the nanoscale in human erythrocytes. Interestingly, we found that CD47 distributes randomly across the erythrocyte membrane as monomers. Using secondary antibody-induced crosslinking, we further found that CD47 exhibits a self-limiting clustering capability compared to the fully mobile CD59 and the cytoskeleton-bound glycophorin C (GPC). Moreover, we reported a decrease in the clustering capability of CD47 in aged cells, thus pointing to a possible new mechanism for erythrocyte senescence.

## Results

### STORM unveils the nanoscale organization of CD47 in human erythrocytes

**Figure 1:**
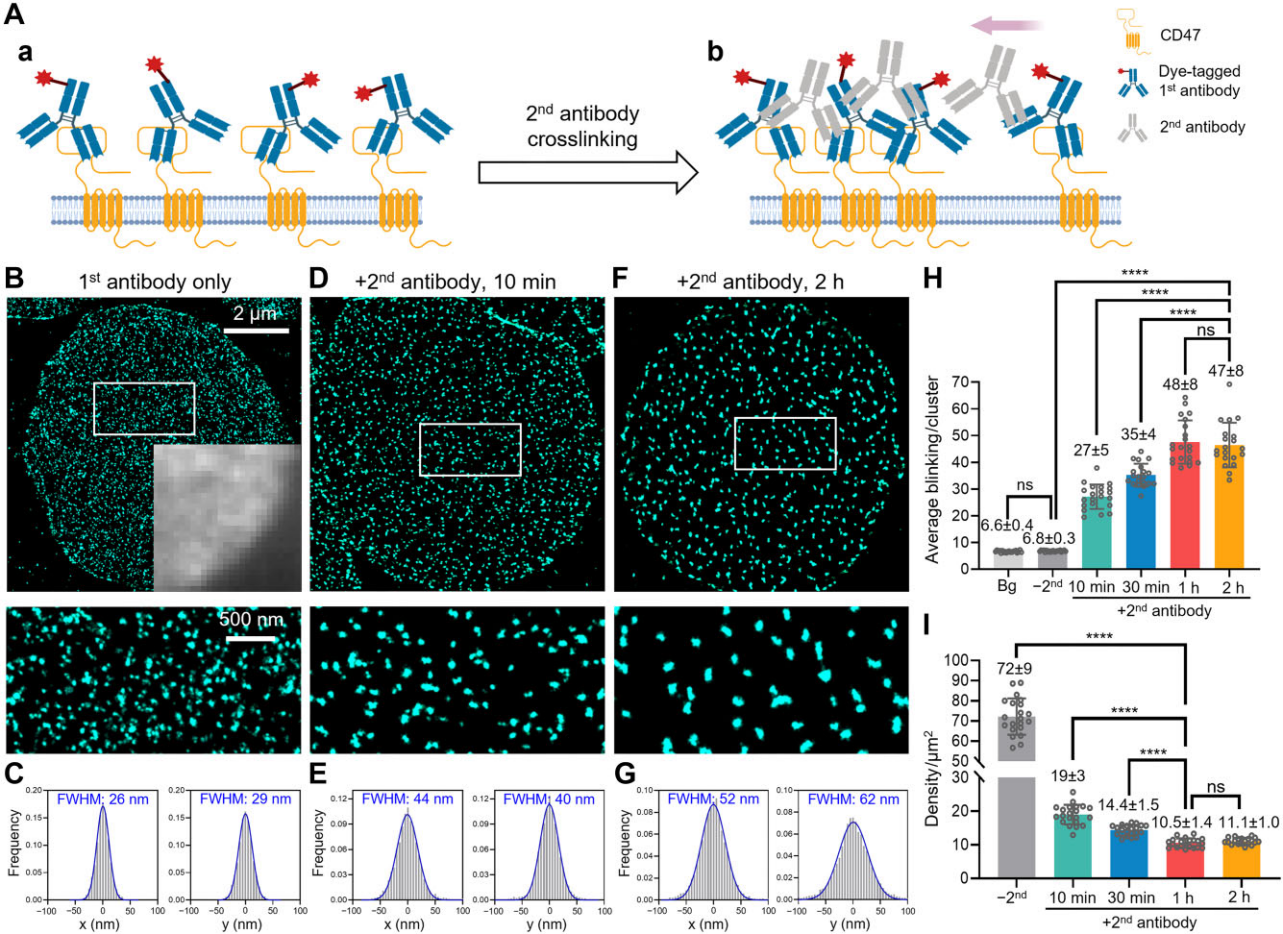
STORM unveils the nanoscale distribution of CD47 and its induced clustering in the erythrocyte membrane. (**A**) Labeling the CD47 extracellular domain with a dye-tagged monoclonal primary antibody (**a**) and subsequent induction of crosslinking/clustering using an unlabeled secondary antibody (**b**). (**B**) STORM image of the primary antibody-labeled CD47 at the bottom surface of an erythrocyte (top) and zoom-in of the white box (bottom). Inset: Diffraction-limited epifluorescence image of a corner of the cell. (**C**) Distributions of single-molecule localizations in the x and y directions after overlaying different clusters in the STORM images in **B** by their centers. Blue lines: Fits to the normal distribution, with resultant FWHM values marked in each plot. **(D**–**G)** STORM images (**D** and **F**) and resultant distributions of single-molecule localizations of overlaid clusters (**E** and **G**) for two other samples after the application of the secondary antibody at 37°C for 10 min (**D** and **E**) and 2 h (**F** and **G**), respectively. (**H**) The average counts of single-molecule blinking events detected for each cluster labeled by the CD47 primary antibody, for erythrocytes not crosslinked by the secondary antibody (−2^nd^) and crosslinked by the secondary antibody at 37°C for different durations (+2^nd^), compared to that for isolated single antibodies on the coverslip (Bg). Each data point corresponds to the average count from ∼100 clusters in one cell. (**I**) Number of STORM-resolved CD47 clusters per unit area of the erythrocyte membrane, for samples without (−2^nd^) or with (+2^nd^) the secondary antibody treatment for different durations. Each data point corresponds to the result from one cell.

Fresh human erythrocytes were immunolabeled with a dye-tagged monoclonal primary antibody that recognized the extracellular domain of CD47 (B6H12 clone) (Figure 1A, left). The labeled cells were adhered to a polylysine-coated coverslip and chemically fixed before being mounted in STORM imaging buffer. At the conventional spatial resolution of ∼300 nm, diffraction-limited fluorescence microscopy images (Figure 1B, inset) did not resolve meaningful structures or distributions.

In contrast, at ∼20-nm spatial resolution, STORM combined with a developed density-based spatial clustering of applications with noise (DBSCAN) algorithm (Supplementary Figure S1) clearly resolved the nanoscale distribution pattern of CD47 (Figure 1B). Interestingly, while distributed at a high density across the membrane, the CD47 labeling appeared as well-separated clusters of random appearances. Overlaying the localized single molecules from different clusters by their centers showed normal distributions with standard deviations of ∼12 nm and a full width at half maximum (FWHM) of ∼25 nm in both the horizontal and vertical directions (Figure 1C), comparable to the resolution limit of STORM (Rust et al., 2006; Huang et al., 2008). The average count of single-molecule blinking events in each CD47 cluster in the erythrocyte membrane (6.8 ± 0.3) was nearly identical to that found for the occasionally observed, isolated single ‘background’ antibodies on the coverslip (6.6 ± 0.4) (Figure 1H). These results suggest that, in this case, each observed ‘cluster’ was due to the repeated photoswitching of a single antibody targeting a single CD47 molecule.

It is well accepted that tropomodulin (TMOD) and the N-terminus of β-spectrin, both located at the actin junctions of the spectrin–actin cytoskeleton network, exhibit a quasi-triangular ∼80-nm lattice structure (Lux, 2016; Pan et al., 2018). As expected, these two reference targets with lattice patterns showed a relatively uniform distribution by analyzing the nearest-neighbor distance, two-dimensional autocorrelation, and Voronoï diagram based on the STORM data (Supplementary Figure S2). However, CD47 was less ordered and randomly distributed (Supplementary Figure S2). The number of STORM-resolved CD47 clusters per unit area of the membrane was 72 ± 9 clusters/μm^2^ (Figure 1I). Considering the typical surface area of an erythrocyte to be ∼150 μm^2^ (Guest, 1948) and our assumption that each cluster corresponds to a single copy of CD47, this result gives a total count of ∼1.1 × 10^4^ CD47 molecules in an erythrocyte, which is in line with previous estimations (∼1.7 × 10^4^) based on biochemistry (Lux, 2016).

Together, our results show that CD47 distributes randomly as monomers across the erythrocyte membrane.

### STORM unveils contrasting clustering capabilities of CD47, CD59, and GPC in the human erythrocyte membrane

**Figure 2:**
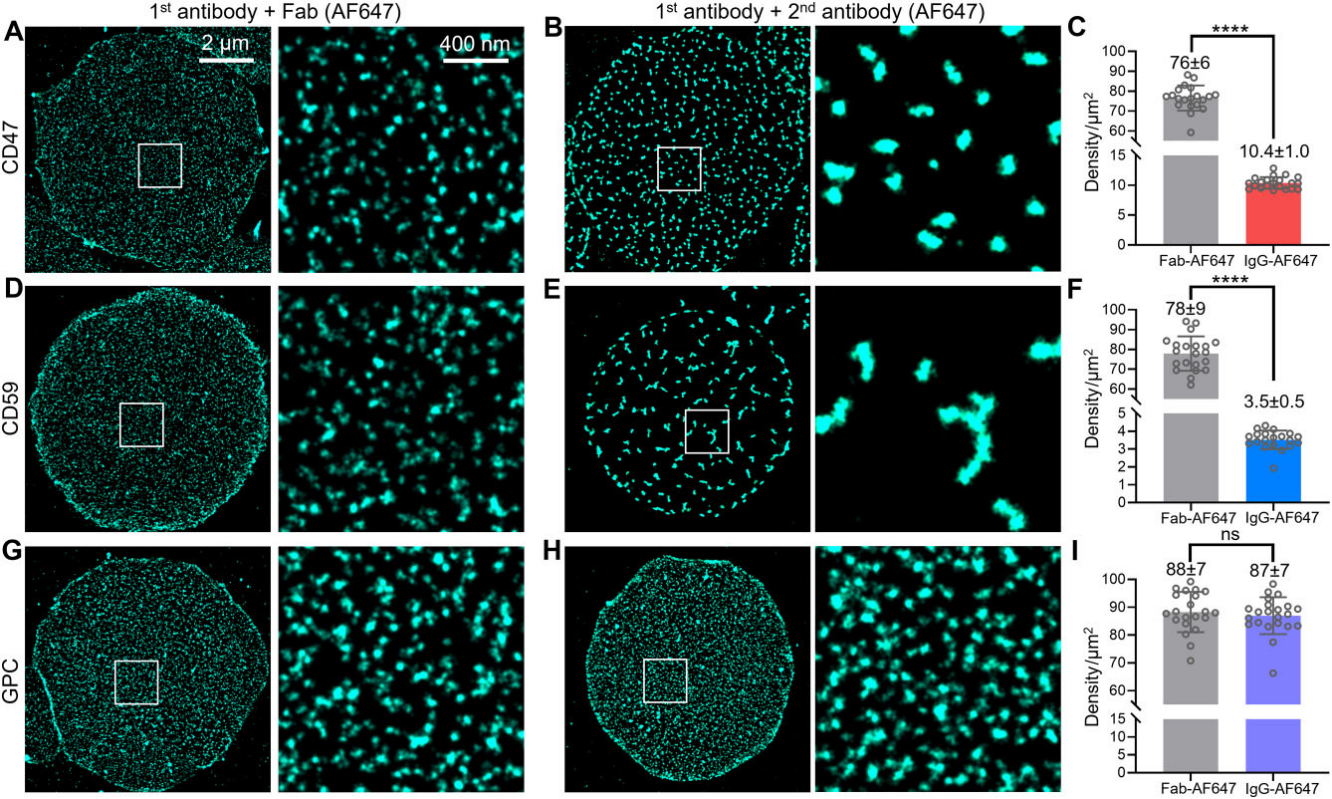
STORM unveils contrasting clustering capabilities of CD47, CD59, and GPC in the erythrocyte membrane. (**A** and **B**) Representative STORM images of CD47 at the bottom surface of erythrocytes (left) and zoom-in of the white boxes (right), first labeled with an untagged anti-CD47 primary antibody, and then followed by a dye-tagged Fab (**A**) or a dye-tagged secondary antibody (**B**) with 1-h incubation at 37°C, respectively. (**C**) Statistics of the number of STORM-resolved CD47 clusters per unit area of the membrane. Each data point corresponds to the result from one cell. (**D**–**F**) Similar to **A**–**C**, but using a primary antibody targeting the extracellular domain of CD59. (**G**–**I**) Similar to above, but using a primary antibody targeting the extracellular domain of GPC.

Interestingly, as we next compared the STORM results in which erythrocytes were labeled with the same anti-CD47 monoclonal antibody but followed by a dye-tagged secondary antibody for indirect immunofluorescence, we observed drastically different patterns (Figure 2B). The number of clusters in each cell was substantially smaller, and each cluster appeared larger with more counts of single-molecule blinking (Figure 2B). These results suggested that the secondary antibody induced CD47 clustering.

To scrutinize this possibility, we first labeled the cells with a dye-tagged anti-CD47 primary antibody and then incubated the sample with an unlabeled secondary antibody for different durations (Figure 1A, right). We found notable changes in the STORM results after a 10-min incubation at 37°C (Figure 1D), with the number of clusters dropped dramatically from 72 ± 9 clusters/μm^2^ to 19 ± 3 clusters/μm^2^ (Figure 1I). Meanwhile, the apparent sizes of the clusters increased substantially, with the FWHM increasing from ∼25 nm to ∼42 nm (Figure 1E), indicating the formation of nanoscale domains larger than the STORM detection limit. The average count of single-molecule blinking events in each cluster also increased markedly to 27 ± 5 (Figure 1H), suggesting ∼4 copies (27/6.6) of CD47 in each cluster. Multiplying this value by the reduced density of 19 clusters/μm^2^ yielded ∼76 copies/μm^2^ of CD47, close to the ∼72 copies/μm^2^ estimated above when the secondary antibody was not applied. This finding suggested that, as CD47 clustered together on the cell membrane, its total amount remained relatively constant.

A longer incubation led to further clustering (Figure 1F and G), yet this process stabilized after ∼1 h. Thus, similar cluster area densities of 11 ± 1 clusters/μm^2^ were observed for samples incubated with the secondary antibody for 1 h and 2 h (Figure 1I). The average counts of single-molecule blinking events were also similar for both conditions (Figure 1H), translating to ∼7 copies of CD47 in each cluster. This number again matched well with the fold decrease in the area densities of clusters, indicating that a fixed total number of CD47 molecules (∼77 copies/μm^2^) aggregated into fewer clusters over time. Additional experiments showed that clustering occurred more slowly at room temperature and 4°C (Supplementary Figure S3), suggesting that the process is driven by the mobility of CD47 in the plasma membrane.

Further experiments showed that substituting the full immunoglobulin G (IgG) secondary antibody with a dye-tagged monovalent fragment (Fab) for indirect immunofluorescence yielded STORM results comparable to those obtained by direct immunofluorescence. The staining signals exhibited a random distribution at an area density of 76 ± 6 clusters/μm^2^ (Figure 2A and C). In contrast, staining with the dye-tagged full IgG secondary antibody led to significant clustering that stabilized to 10.4 ± 1.0 clusters/μm^2^ (Figure 2B and C), comparable to what we observed above when the unlabeled secondary antibody was applied to samples labeled with the dye-tagged primary antibody (Figure 1F and I).

Taken together, these findings indicate that CD47 is distributed as individual molecules in the erythrocyte membrane but can be readily clustered by full IgG secondary antibody crosslinking, converging to a fixed final density of ∼11 clusters/μm^2^.

To further understand the clustering capability of CD47, we next compared two other abundant proteins on the erythrocyte membrane, CD59 and GPC. CD59 is a glycosylphosphatidylinositol (GPI)-anchored protein that freely diffuses in the membrane (Discher et al., 1994), whereas GPC is bound to the cytoskeleton and thus is immobile (Dahl et al., 2003). Both proteins contain extracellular domains that can be immunolabeled as with CD47.

STORM of CD59 labeled with a primary antibody and dye-tagged Fab showed a random distribution similar to that of CD47 (Figure 2D), and the density was also comparable at ∼80 clusters/μm^2^ (Figure 2F). Notably, when the full IgG secondary antibody was used, dramatic aggregation occurred on the plasma membrane, so that sparsely distributed domains were formed with curious dendritic shapes up to ∼500 nm in length (Figure 2E). Meanwhile, the number of domains across the cell reduced drastically to ∼3 domains (clusters)/μm^2^ (Figure 2F). In contrast, comparable STORM results were obtained for GPC labeled with a primary antibody and then labeled with either the dye-tagged Fab or the full IgG secondary antibody. No clustering was induced by the secondary antibody, and the same cluster density of ∼88 ± 7 clusters/μm^2^ was found for both conditions (Figure 2G–I). Cluster area and Ripley's K analysis also yielded contrasting results for the three proteins, which is consistent with the above discussion (Supplementary Figure S4).

The above results suggest that CD47 has an intermediate clustering capability between that of the freely diffusing GPI-anchored CD59 and the cytoskeleton-bound GPC. The finding that CD47 did not form extensive domains like CD59 but instead stabilized at a fixed number of clusters on the cell membrane (i.e. self-limiting clustering) suggests that CD47 contains an immobile fraction possibly bound to the underlying cytoskeleton. Interestingly, Voronoï diagram analysis of the secondary antibody-induced CD47 clusters revealed a uniform distribution (Supplementary Figure S5), similar to that of cytoskeleton proteins, supporting a potential association of these CD47 clusters with the cytoskeleton.

### Two-color STORM co-labeling and co-clustering experiments further elucidate the fractional cytoskeleton attachment of CD47

**Figure 3:**
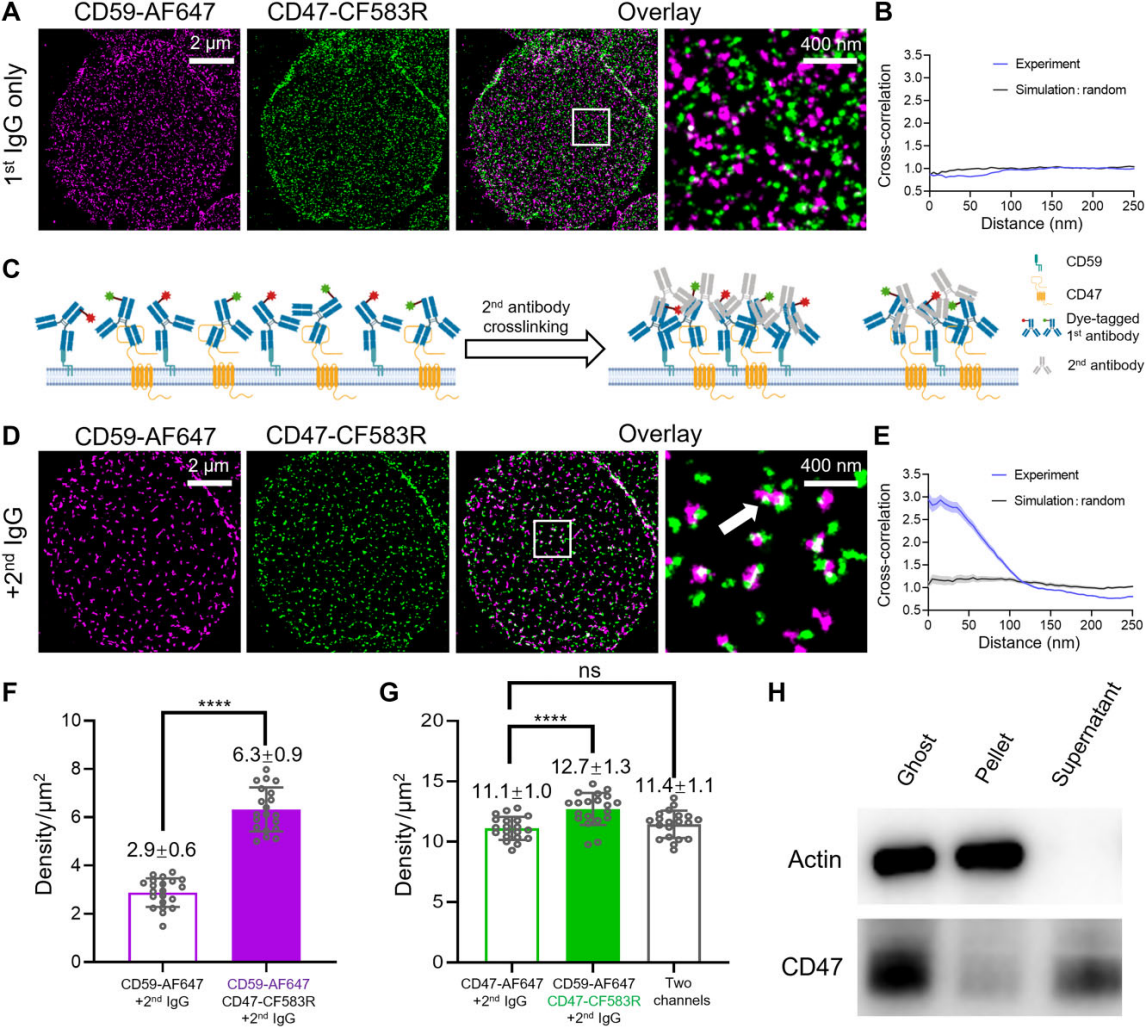
Two-color STORM elucidates fractional cytoskeleton attachment of CD47. (**A**) Representative two-color STORM image of the bottom surface of an erythrocyte co-labeled by two primary antibodies, respectively, targeting the extracellular domains of CD59 (tagged with AF647) and CD47 (tagged with CF583R). Images are shown as separate channels, overlay, and zoom-in. (**B**) Spatial cross-correlation between the two-color channels for a 3 × 3-μm^2^ region of the data (blue), versus a simulated curve for random distributions (gray). (**C**) Model: Crosslinking the labeled anti-CD47 and anti-CD59 primary antibodies with an unlabeled secondary antibody. (**D**) Representative two-color STORM image of another sample similarly labeled as in **A** but after incubation with the secondary antibody at 37°C for 2 h. (**E**) Spatial correlation between the two-color channels for a 3 × 3-μm^2^ region of the data (blue), versus a simulated curve for random distributions (gray). (**F**) Statistics of the count of STORM-resolved CD59 clusters per unit area after secondary antibody crosslinking, for cells without and with the co-incubation of the anti-CD47 antibody. Each data point corresponds to the average count from one cell. (**G**) Statistics of the number of STORM-resolved CD47 clusters per unit area after secondary antibody crosslinking, for samples without and with the co-incubation of the anti-CD59 antibody, as well as when the merged two-color STORM image of anti-CD47 and anti-CD59 is used for the cluster analysis. (**H**) Immunoblot data of Triton-treated erythrocyte ghosts for actin and CD47, for the total amount, the pellet (attached to the cytoskeleton), and the supernatant (unbound to the cytoskeleton), respectively.

To further examine the fractional cytoskeleton attachment behavior of CD47, we devised an experiment in which, in the same sample, CD47 and CD59 were first co-labeled with two primary monoclonal antibodies tagged with two spectrally distinct dyes. Two-color STORM showed that the two targets each appeared as random clusters across the erythrocyte membrane (Figure 3A), comparable to what we observed when they were individually labeled. Spatial cross-correlation between the two channels indicated no significant co-localization or exclusion (Figure 3B), suggesting that the two proteins do not interact natively.

We next applied a secondary antibody to crosslink both primary antibodies (Figure 3C). Two-color STORM (Figure 3D) showed that CD59 exhibited markedly more restrained clustering than when the CD47 primary antibody was not applied. Large dendritic domains were no longer generated, and the number of clusters visibly increased, with quantification showing a rise from 2.9 ± 0.6 clusters/μm^2^ to 6.3 ± 0.9 clusters/μm^2^ (Figure 3F). In comparison, STORM of the same sample showed less change in the clustering behavior of CD47 (Figure 3D) when compared to samples without the CD59 antibody, with quantification showing a slight increase in the number of clusters from 11.1 ± 1.0 clusters/μm^2^ to 12.7 ± 1.3 clusters/μm^2^ (Figure 3G). Overlaying the two-color STORM images showed substantial co-localization of the CD59 and CD47 clusters (Figure 3D). Accordingly, spatial cross-correlation between the two channels indicated strong co-localization with spatial shifts below ∼100 nm (Figure 3E).

The above results indicate that CD59 became less mobile as it was locally tied to the immobile fraction of CD47 that defined the number of CD47 clusters in the final state. However, the clustering capability of CD47 was minimally affected by the presence of the CD59 labels. Still, CD59 labels generated many ∼100-nm linear domains, and in some cases, CD47 bound to different parts of the same domain was resolved as different clusters in the CD47 channel (arrow in Figure 3D), thus explaining the slight increase in the count of CD47 clusters. Merging the two-color STORM images into a single channel to count the final number of clusters overcame this issue, recovering the typical ∼11 clusters/μm^2^ values of CD47 clusters when the CD59 antibody was not applied (Figure 3G).

Therefore, our two-color STORM results confirmed that a fraction of the CD47 proteins are locally bound to the underlying cytoskeleton, while the rest diffuses in the membrane. Besides, immunoblots of Triton-treated erythrocytes have been used to qualitatively investigate the cytoskeleton attachment of membrane proteins (Mouro-Chanteloup et al., 2003). By applying similar methods, we observed a small fraction of CD47 remained bound to the cytoskeleton (Figure 3H), consistent with the results obtained by combining STORM and secondary antibody-induced crosslinking.

To study the potential interactions between CD47 and the membrane cytoskeleton, we performed two-color STORM for CD47 versus actin or ankyrin (Supplementary Figure S6), as well as co-localization simulation (Supplementary Figure S7). For samples not crosslinked by secondary antibodies, we thus observed no noticeable correlations between the labeled CD47 and actin or ankyrin, suggesting that most CD47 molecules do not directly interact with the cytoskeleton (Supplementary Figures S6 and S7). Upon secondary antibody crosslinking, we observed enhanced co-localization of the CD47 clusters with ankyrin but not with actin (Supplementary Figures S6 and S7), with the caveat that the enlarged CD47 cluster sizes complicated the analysis.

### STORM reveals a decrease in both the density and clustering capability of CD47 in stored erythrocytes

**Figure 4:**
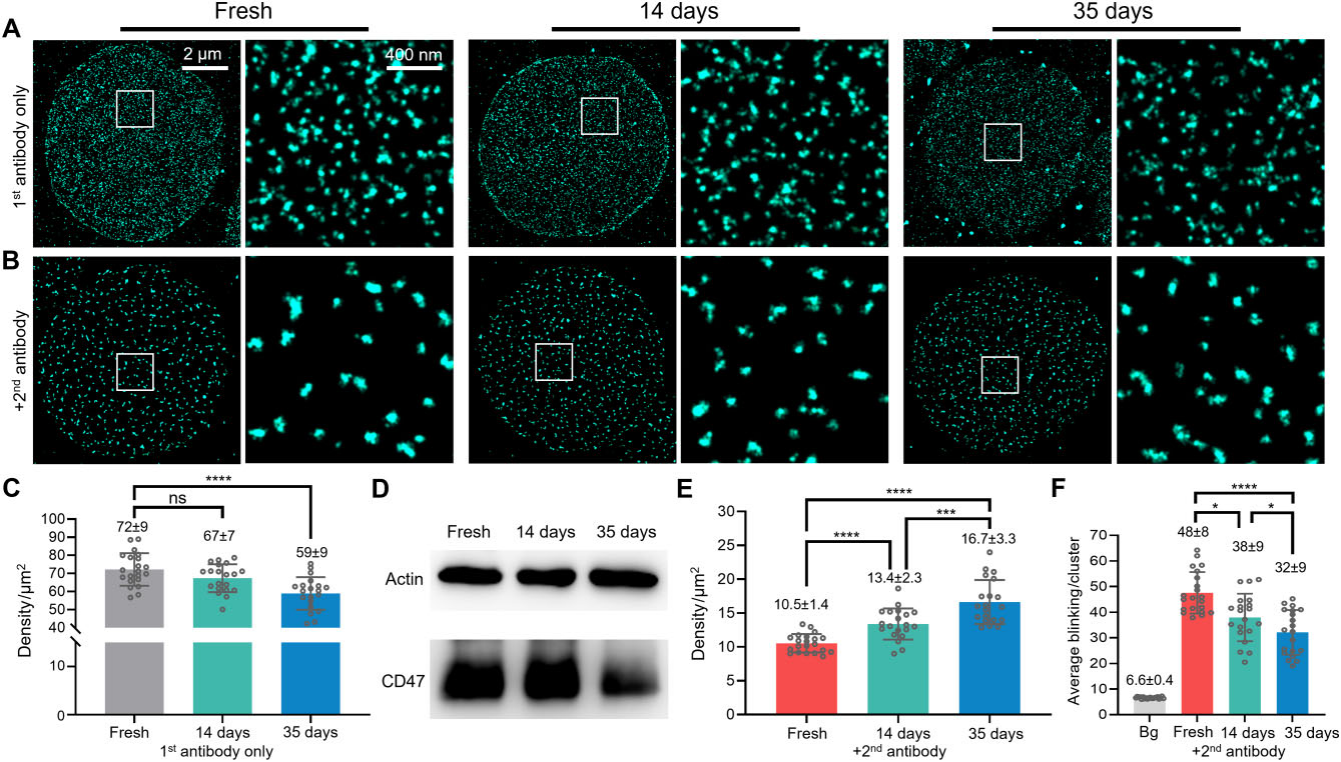
STORM indicates a reduction in the CD47 clustering capability in stored erythrocytes. (**A**) Representative STORM images and zoom-ins of CD47 labeled by the dye-tagged primary antibody, for erythrocytes freshly prepared (left) and stored at 4°C in the SAGM storage solution for 14 (center) days and 35 (right) days, respectively. (**B**) Representative STORM images and zoom-ins for similar samples labeled by the dye-tagged primary antibody, but then incubated with an unlabeled secondary antibody at 37°C for 1 h. (**C**) Statistics of the counts of STORM-resolved CD47 clusters per unit area for the different samples, when only labeled by the dye-tagged primary antibody. (**D**) Immunoblot of total CD47, for cells stored under the same conditions. (**E**) Statistics of the counts of STORM-resolved CD47 clusters per unit area for samples that have been crosslinked with the secondary antibody. (**F**) Distribution of the average single-molecule blinking counts per cluster.

As CD47 is implicated in mechanisms for the immune system to detect and eliminate senescent erythrocytes (Khandelwal et al., 2007; Oldenborg, 2012; Thiagarajan et al., 2021), we next examined how the density and self-limiting clustering capability of CD47 evolve during the aging process of human erythrocytes stored in the standard saline–adenine–glucose–mannitol (SAGM) solution at 4°C. STORM imaging of samples labeled with the dye-tagged primary antibody against CD47 (Figure 4A) showed comparable results between freshly prepared samples and samples stored for 14 days and a small drop in CD47 cluster density in samples stored for 35 days. Statistical analysis revealed a ∼15% reduction in cluster density per unit area, decreasing from ∼70 clusters/μm^2^ to ∼60 clusters/μm^2^ (Figure 4C). Immunoblot results also showed a mild drop in CD47 expression levels after 35 days of storage (Figure 4D), consistent with the STORM results. The average count of single-molecule blinking events of CD47 in fresh, 14-day storage, and 35-day storage erythrocytes was nearly identical to that of isolated single ‘background’ antibodies on the coverslip (Supplementary Figure S8), suggesting that CD47 maintained monomers in aged erythrocytes. Additionally, using 2D3 clone CD47 primary antibodies (Burger et al., 2012), which selectively bind to oxidized CD47 in aged erythrocytes in comparison with fully labeling B6H12 clone antibodies, we further investigated the conformational changes of CD47. STORM results showed that more CD47 transformed into the oxidized conformation during storage (7.4 ± 1.9/μm^2^ for fresh cells vs. 15.6 ± 4.3/μm^2^ for 35-day cells) (Supplementary Figure S9).

Interestingly, as we applied the secondary antibody to induce clustering, the samples with longer storage times ended up with higher cluster densities per unit area (Figure 4B and E), even though the cluster density was lower for the older cells when the secondary antibody was not applied. At the same time, single-molecule blinking statistics indicated that the average count of CD47 molecules in each secondary antibody-induced cluster dropped from ∼7.3 in the fresh erythrocyte sample to ∼4.8 in erythrocytes after 35-day storage (Figure 4F). Together, through STORM combining secondary antibody-induced crosslinking, these results suggest that not only the total amount of CD47 (Figure 4A and C), but also their clustering capabilities decreased in aged erythrocytes (Figure 4B–F).

## Discussion

By achieving ∼20-nm spatial resolution with STORM and further quantifying single-molecule blinking counts, we showed with dye-tagged primary antibodies that CD47 was distributed randomly as individual molecules across the erythrocyte membrane (Figure 1B), with the ∼75 copies/μm^2^ in general agreement with the expected amount of CD47 in the erythrocyte (Lux, 2016). Early studies indicated that CD47 formed micro-clusters in cancer cells and changed to a diffuse pattern during apoptosis (Lv et al., 2015). In mouse erythrocytes, CD47 is found to reside as clusters, which become larger and denser during aging (Wang et al., 2020). Quite differently, we demonstrated that, in native human erythrocytes, CD47 existed as monomers, rather than clusters on the membrane.

Through STORM, we next found that the application of a secondary antibody induced fast clustering/aggregation of the labeled CD47 in the membrane (Figure 1D). Comparison with the results of the GPI-anchored CD59 and cytoskeleton-bound GPC further suggested that CD47 was partially mobile. Under strong crosslinking conditions, the system stabilized toward ∼11 clusters/μm^2^ on the cell membrane with an average count of ∼7 CD47 molecules in each cluster (Figure 1H and I). Antibody-induced crosslinking has been previously employed to unveil the relationship between membrane proteins and the cytoskeleton by fluorescence microscopy (Huet et al., 1980). In the present work, STORM combined with secondary antibody-induced crosslinking suggests the cytoskeleton attachment rate of CD47 as ∼14% (from ∼76 clusters/μm^2^ to ∼11 clusters/μm^2^ by secondary antibody-induced crosslinking, as shown in Figure 2C), so that in samples co-labeled with anti-CD47 and anti-CD59 antibodies, the secondary antibody-induced clustering capability of CD59 was substantially reduced (Figure 3).

Discher et al. proposed the FIMD method to evaluate the cytoskeleton attachment rates for erythrocyte membrane proteins (Discher et al., 1994). A micropipette is employed to aspirate a part of the erythrocyte, generating a local deformation, and the fluorescence intensity distribution along the deformed membrane is used to estimate the mobility of different membrane proteins. The cytoskeleton-labeled erythrocyte ghosts show smooth and axisymmetric deformation over the entire cell, whereas with the fully mobile CD59, the fluorescence intensity increases progressively toward the tip of the projection (Discher et al., 1994). Using these two references, FIMD estimates an ∼60% fractional attachment for CD47 in the human erythrocyte (Dahl et al., 2003). However, assumptions need to be made in interpreting the complex FIMD signal, and the severe membrane deformation involved may further alternate the intrinsic behaviors of the membrane proteins.

Moreover, previous studies suggested possible co-localization of CD47 with other ankyrin-associated proteins, including RhAG through immunofluorescence (Dahl et al., 2003), Protein 4.2 via gene deficiency (Bruce et al., 2002, 2003; Mouro-Chanteloup et al., 2003; Dahl et al., 2004), and Band 3 protein via co-immunoprecipitation (Bruce et al., 2003), indicating that CD47 located at the ankyrin complex. Our two-color STORM results exhibited a strong visible co-localization of clustered CD47 and ankyrin (Supplementary Figures S6 and S7), suggesting that immobile CD47 interacts with the ankyrin complex, which leads to the self-limiting clustering capability of CD47.

The decrease in CD47 amount is a key indicator of erythrocyte aging that triggers phagocytosis by macrophage (Khandelwal et al., 2007; de Back et al., 2014). The potential changes in CD47 during erythrocyte aging have been controversial, with contradicting studies suggesting large (∼50%, detected by enzyme-linked immunosorbent assay) (Stewart et al., 2005) or small (∼5%, detected by flow cytometry) (Anniss and Sparrow, 2002) decreases in the CD47 level over ∼1-month storage. With STORM, we obtained an ∼15% decrease in the CD47 level on the membrane (from 73 clusters/μm^2^ to 59 clusters/μm^2^, as shown in Figure 4C). Notably, both being detected by flow cytometry in erythrocytes stored for 35 days, CD47 in mice erythrocytes without skeleton binding (Subramanian et al., 2006) had a faster decline rate, ∼40% for mice erythrocytes (Gilson et al., 2009) versus ∼5% for human erythrocytes (Anniss and Sparrow, 2002). Thus, binding of CD47 to the skeleton might inhibit the loss of CD47 during aging.

On the other hand, previous studies showed that CD47 in mouse was fully lateral mobile without cytoskeleton attachment (Subramanian et al., 2006), different from human erythrocytes as suggested in our study. It is known that mouse erythrocytes have a 40-day lifespan (Van Putten and Croon, 1958), whereas human erythrocytes can survive for 120 days (Shemin and Rittenberg, 1946; Coffey and Ganz, 2017). Thus, the lifespan of erythrocytes may be associated with the cytoskeleton attachment rate of the immune checkpoint protein CD47. In contrast, for cancer cells, CD47 was found to distribute as micro-clusters, while CD47 clusters diffused during cell apoptosis induced by ultraviolet irradiation, resulting in the clearance by macrophages (Lv et al., 2015). Wang et al. (2020) reported that, on young mouse erythrocytes, CD47 resided as clusters, while on aged cells, CD47 decreased in number but formed bigger clusters.

Interestingly, it has been suggested that a relatively low density of CD47 is sufficient to inhibit phagocytosis (Tsai et al., 2010). Recent conventional and super-resolution microscopy results have shown that SIRPα is organized in discrete clusters in macrophages (Lopes et al., 2017), implying that CD47 interacts with SIRPα in the form of clusters to activate the ‘don't eat me’ signaling pathway. Previous studies reported that CD47 formed clusters upon binding to SIRPα-coated atomic force microscope tips (Subramanian et al., 2006). Further co-culture experiments of erythrocytes and THP-1-derived macrophages showed that, on the cell–cell conjugate sites, CD47 formed large clusters (Supplementary Figure S10), indicating that the clustering capability of CD47 is crucial for keeping erythrocytes from immune clearance. Therefore, together with other recent studies highlighting the functional roles of the clustering of membrane proteins (Cebecauer et al., 2010; Ha et al., 2013; Pageon et al., 2013; Garcia-Parajo et al., 2014; Hu et al., 2016; Lopes et al., 2017; Platre et al., 2019; Pan et al., 2020), we point to a possible new insight of CD47–SIRPα interaction. In healthy erythrocytes, sufficient CD47 aggregate into clusters upon interaction with SIRPα in macrophages to avoid phagocytosis. In aged erythrocytes, CD47 with low density and frustrated clustering capability is less effective in activating the CD47–SIRPα-mediated ‘don't eat me’ signaling pathway, thus leading to clearance by macrophages.

Besides, a previous study reported a reduction of lipid raft during erythrocyte aging (Salzer et al., 2008). Treating erythrocytes with methyl-β-cyclodextrin (5 mM) to disrupt cholesterol-enriched lipid raft (Cai et al., 2012; Sezgin et al., 2017), we showed a significant decrease of CD47 density (Supplementary Figure S11), implying that the decreased CD47 amount is associated with lipid raft loss on aged erythrocytes.

In summary, with the outstanding spatial resolution and single-molecule quantification capability afforded by STORM combining secondary antibody-induced crosslinking, we have established a new strategy to quantify both the nanoscale organization and clustering capabilities of CD47 in human erythrocytes. We thus found that CD47 randomly distributed as monomers across the membrane in the native cell, yet exhibited good clustering capability towards the ∼14% cytoskeleton-anchored fraction. Moreover, we showed that, during erythrocyte storage, CD47 exhibited modest decreases in the total amount and steady drops in its clustering capability. Such diversities in CD47 behavior may play functional roles in how CD47 acts as a ‘don't eat me’ signal. The combination of STORM and secondary antibody-induced crosslinking developed in this work also opens a new door to quantitatively investigate the cytoskeleton-attachment behavior of diverse membrane proteins at the nanoscale.

## Materials and methods

### Reagents

Poly-l-lysine (pLL; molecular weight: 70–150 kDa) solution (P4707), saponin (S4521), bovine serum albumin (BSA; A3059), cysteamine (30070), glucose oxidase (G2133), catalase (C30), Triton X-100 (T8787), d-(+)-glucose (G7528), and other general reagents were from Sigma-Aldrich. EM-grade paraformaldehyde (15714) and glutaraldehyde (16365) were from Electron Microscopy Sciences. Alexa Fluor 647-conjugated phalloidin (A22287) was from Invitrogen. Electrochemiluminescence (ECL) detection reagents were from Proteintech.

The primary antibodies used were as follows: anti-CD47 (B6H12), mouse monoclonal (sc-12730, Santa Cruz Biotechnology; or BE0019-1, Bio X Cell); Alexa Fluor 647-conjugated anti-CD47 (B6H12), mouse monoclonal (sc-12730 AF647, Santa Cruz Biotechnology); anti-GPC (BRIC10), mouse monoclonal (sc-59183, Santa Cruz Biotechnology); anti-CD59 (MEM-43), mouse monoclonal (MA1-19133, Invitrogen); anti-β-actin, mouse monoclonal (60008-1-Ig, Proteintech); anti-TMOD, mouse monoclonal (TA503146, OriGene); anti-N-terminus of β-spectrin (actin-binding domain), rabbit polyclonal (ABT185, Millipore); and anti-ankyrin, mouse monoclonal (ab212053, Abcam).

The secondary antibodies used were as follows: AffiniPure donkey anti-mouse IgG (H+L) (715-005-151, Jackson ImmunoResearch); Alexa Fluor 647 AffiniPure donkey anti-mouse IgG (H+L) (715-605-151, Jackson ImmunoResearch); horseradish peroxidase-conjugated anti-mouse secondary antibody (A0216, Beyotime); AffiniPure donkey anti-rabbit IgG (H+L) (711-005-152, Jackson ImmunoResearch); AffiniPure goat anti-mouse IgG_2b_ (115-005-207, Jackson ImmunoResearch); and AffiniPure goat anti-mouse IgG_1_ (115-005-205, Jackson ImmunoResearch).

The secondary antibody Fab fragment used was Alexa Fluor 647 AffiniPure Fab fragment goat anti-mouse IgG (H+L) (115-607-003, Jackson ImmunoResearch).

The following antibodies were used for dye-tagging: anti-CD47 (BE0019-1, Bio X Cell) conjugated with CF583R succinimidyl ester (#96084, Biotium) and anti-CD59 (MA1-19133, Invitrogen) conjugated with Alexa Fluor 647 succinimidyl ester (A37573, Invitrogen).

### Sample preparation

Acid-washed 12-mm glass coverslips were coated with 0.1-mg/ml pLL for 3 h at room temperature. Then, they were washed with deionized water and dried before being placed in 24-well plates. Fresh human fingertip blood was diluted in phosphate buffered saline (PBS) containing 10 mM glucose and 10-mg/ml BSA (PBS-GB), centrifuged twice at 700× *g*, and resuspended in PBS-GB to obtain an erythrocyte suspension at ∼5 × 10^5^ cells/ml. For the storage of erythrocytes, blood was diluted in a SAGM additive solution (H20045599, NIGALE) at the same concentration and stored at 4°C for 14 days or 35 days. For direct immunofluorescence with dye-tagged primary antibodies, the erythrocyte suspension was incubated with the primary antibodies (anti-CD47-AF647, anti-CD47-CF583R, and anti-CD59-AF647) for 1 h at room temperature. The samples were centrifuged twice to remove the residual dye-tagged primary antibodies and resuspended in 500 μl of PBS-GB. Then, the cell suspension was added to a prepared 24-well plate with pLL-coated glass coverslips for cell adherence for 30 min at room temperature (Figures 1B, 3A, and 4A). Experiments on the secondary antibody-induced aggregation of primary antibodies were carried out in two ways. In one experiment, the erythrocyte suspension was first labeled with dye-tagged primary antibodies (anti-CD47-AF647, anti-CD47-CF583R, and anti-CD59-AF647), and then centrifuged twice to remove the residual primary antibodies. The samples were then incubated with untagged secondary antibodies [donkey anti-mouse IgG (H+L); 715-005-151, Jackson ImmunoResearch] (Figures 1D and F, 3D, and 4B). In the other experiment, erythrocyte suspension was incubated with untagged primary antibodies (anti-CD47, anti-CD59, and anti-GPC), centrifuged twice to remove excess primary antibodies, and then labeled with dye-tagged secondary antibodies [Alexa Fluor 647 donkey anti-mouse IgG (H+L); 715-605-151, Jackson ImmunoResearch] (Figure 2). After the incubation with the secondary antibodies, the samples were centrifuged twice and resuspended in PBS-GB. The resuspended erythrocytes were placed in a 24-well plate with pLL-coated glass coverslips and allowed to adhere as described previously. The attached cells were treated with 0.0015% saponin in PBS for 5 min, washed with PBS, fixed with 3% paraformaldehyde and 0.1% glutaraldehyde for 15 min, and mounted for imaging.

### STORM

The samples on the 12-mm coverslips were mounted on freshly cleaned 22-mm × 60-mm rectangular glass slides for super-resolution imaging via STORM. The imaging buffer consisted of 5% (*w*/*v*) glucose, 100 mM cysteamine, 0.8-mg/ml glucose oxidase, and 40-μg/ml catalase in Tris-HCl (pH 7.5). The fluorescence images were collected via a home-built STORM setup based on an inverted optical microscope (Ti-E, Nikon) equipped with an electron-multiplying charge coupled device (iXon Ultra 897, Andor), using a 100× oil-immersion objective (Nikon CFI Plan Apochromat λ, numerical aperture = 1.49). A strong excitation laser of 647 nm or 561 nm (∼2 kW cm^−2^) was used to photoswitch most of the dye molecules into a dark state, while also exciting fluorescence from the remaining, sparsely distributed emitting dye molecules in the labeled sample. Using a weak 405-nm laser (typically 0–1 W cm^−2^) concurrently with the 647-nm or 561-nm lasers, fluorophores were reactivated into the emitting state, which allows only a small, optically resolvable fraction of fluorophores to be in the emitting state at any given instant. Images were recorded at 110 frames per second. Typically, ∼40000 frames were recorded per image to determine the location of each molecule using the localization algorithm as described previously (Rust et al., 2006; Huang et al., 2008).

### Western blotting

Fresh human blood (100 μl) was placed in 10 ml of PBS-GB and centrifuged at 700× *g* for 10 min. Afterwards, 3 ml of precooled PBS was added to the pellets, followed by centrifugation twice at 700× *g* for 5 min. Erythrocytes were lysed in 0.0015% saponin solution for 30 min and then centrifuged at 10000× *g* for 10 min to extract membrane proteins. For nonionic detergent solubilization, 1% Triton X-100 (150 μl) was added to the packed ghost in PBS (50 μl) for 2 h at room temperature. Separation of the supernatants and pellets was achieved by centrifugation at 14000× *g* for 15 min at 4°C. Then, the pellets were resuspended in PBS. Equal volumes of pellet fractions and supernatant were subjected to 10% sodium dodecyl sulphate-polyacrylamide gel electrophoresis and transferred to a nitrocellulose membrane. The membrane was blocked for 1 h with 5% skimmed milk at room temperature and immunoblotted overnight at 4°C with anti-CD47 and anti-β-actin antibodies, followed by incubation with a horseradish peroxidase-conjugated secondary antibody. Finally, the ECL detection reagent was added for visualization in a Tanon 5200 MultiImage System.

### Developed DBSCAN algorithm

Clustering analysis of STORM data was performed with a custom MATLAB program using the built-in *dbscan* function. Original point cloud data (Supplementary Figure S1A) were executed coarse clustering by DBSCAN to obtain the preliminary clustering results, followed by refined clustering by combination of DBSCAN and hierarchical clustering algorithms to obtain better-defined results. In detail, a relatively large parameter (ε_1_, M_1_) was selected to perform the DBSCAN calculation for coarse clustering (Supplementary Figure S1B). In this step, the excessively discrete points were removed as noise points; meanwhile, some neighboring points were identified as a cluster. Then, the clusters with an area larger than the average area of all the clusters were chosen for further refined clustering segmentation (Supplementary Figure S1C). For each selected cluster, the M_1_ parameter was kept unchanged, and DBSCAN was executed with a smaller parameter (ε_2_; ε_2_ < ε_1_) for segmentation again. By computing with a loop from ε_2_ to ε_1_, a series of cluster numbers were obtained. The maximum number of clusters was selected as the clustering parameter for hierarchical clustering to be performed. The final clustering segmentation result was obtained from the above two clustering steps.

### Cross-correlation analysis

By calculating the pairwise distances between single molecules identified in two color channels, a two-dimensional cross-correlation analysis was performed (Sengupta et al., 2011; Stone and Veatch, 2015). A histogram was generated using MATLAB to calculate the number of molecule pairs within a range of distances. Several sets of molecules randomly distributed over the same area were generated to normalize this histogram. Thus, in the final cross-correlation curves, in the limit of zero intermolecular distance, patterns in the two channels that are excluded, random, or co-localized should show values of <1, ∼1, or >1, respectively. With increased intermolecular distances, this initial value tends to decay to 1, reflecting the length scale over which exclusions or co-localizations occur. To simulate the STORM data for comparison with cross-correlation analysis, point cloud data of the same area and number of clusters were generated, and the single-molecule localization of each cluster was modeled as a two-dimensional Gaussian distribution with a standard deviation determined according to the radius of the membrane protein point cluster in the actual STORM image.

### Quantification and statistical analysis

All data are presented as the mean ± standard deviation from at least three independent experiments. Statistical comparison between the two groups was carried out using unpaired Student's *t*-test (GraphPad Prism 9). Statistical significance was defined as *P < 0.05, **P < 0.01, ***P < 0.001, and ****P < 0.0001; ns, not significant (*P* > 0.05).

## Supplementary Material

mjae041_Supplemental_File

## Notes

**Author contributions:** L.P. and K.X. conceived the study. L.P. designed the experiments. J.Y. and F.X. performed the experiments. J.Y., F.X., M.H., H.D., J.C., and W.L. analyzed the data. F.H., W.L., and R.Y. contributed to the interpretation of the results. L.P. and K.X. wrote the manuscript. L.P., K.X., and J.X. supervised the work.
