# Supplementary material for: Super-resolution microscopy unveils the nanoscale organization and self-limiting clustering of CD47 in human erythrocytes
Source: J Mol Cell Biol. 2024 Oct 4;16(9):mjae041. doi: 10.1093/jmcb/mjae041 (PMC11992563; doi:10.1093/jmcb/mjae041)
Supplement: mjae041_Supplemental_File [file mjae041_supplemental_file.pdf]

Supplementary material

Super-resolution microscopy unveils the nanoscale organization and self-limiting clustering of CD47 in human erythrocytes

Jianyu Yang[1, †], Fulin Xing[1, †], Fen Hu[1], Mengdi Hou[1], Hao Dong[1], Jiayu Cheng[1], Wan Li[2], Rui Yan[2], Jingjun Xu[1,4], Ke Xu[2*], Leiting Pan[1,3,4,*]

[1]The Key Laboratory of Weak-Light Nonlinear Photonics of Education Ministry, School of Physics and TEDA Institute of Applied Physics, Nankai University, Tianjin 300071, China
[2]Department of Chemistry, University of California, Berkeley, California 94720, USA
[3]State Key Laboratory of Medicinal Chemical Biology, Frontiers Science Center for Cell Responses, College of Life Sciences, Nankai University, Tianjin 300071, China
[4]Shenzhen Research Institute of Nankai University, Shenzhen, Guangdong 518083, China
[†]J.Y. and F.X. contributed equally to this work.
*Correspondence: Leiting Pan, Email: plt@nankai.edu.cn;
Ke Xu, Email: xuk@berkeley.edu

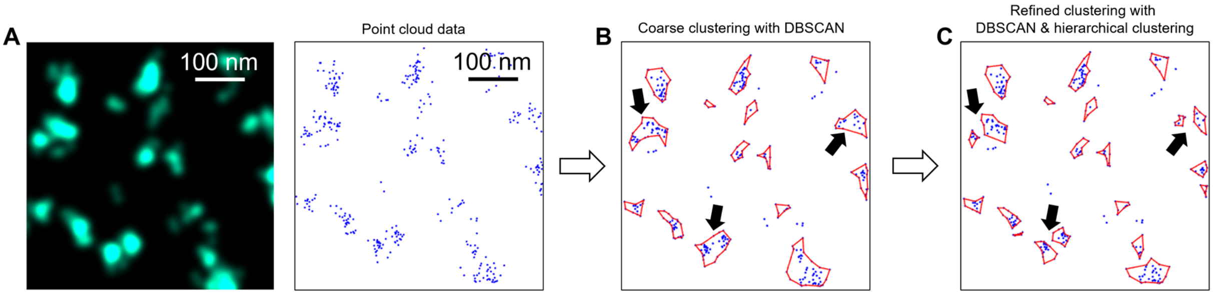

Supplementary Figure S1 DBSCAN-based cluster analysis of CD47 STORM data. (**A**) Representative STORM images of immunolabeled CD47 and corresponding point cloud data without rendering. (**B**) Coarse clustering by DBSCAN. Arrows indicate identified clusters with area larger than the average area of all the clusters. (**C**) Refined clustering by combination with DBSCAN and hierarchical clustering. Arrows indicate better-defined CD47 clusters after refined clustering.

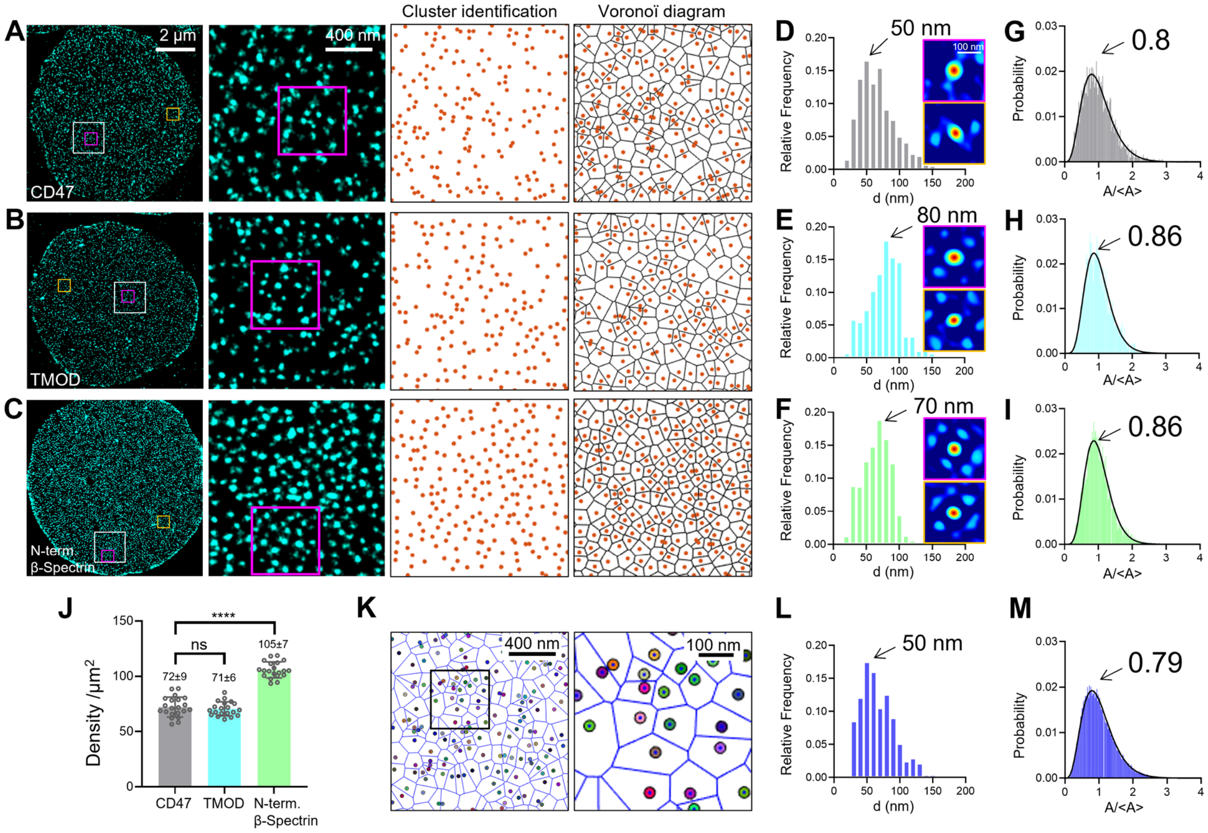

Supplementary Figure S2 Nearest-neighbor distance, two-dimensional autocorrelation and Voronoï diagram analysis of CD47, TMOD, and the N terminus of β-spectrin in erythrocytes. (**A**–**C**) Representative STORM images of immunolabeled CD47 (**A**), TMOD (**B**), the N terminus of β-spectrin (**C**) in single erythrocytes. The white boxes are selected regions, and their corresponding zoomed-in images are shown in the second line. The corresponding scatter plot of the center-of-mass distribution and the Voronoï diagram based on the center-of-mass are shown in the third and fourth lines, respectively. (**D**–**F**) Distributions of distances between nearest neighbors of CD47 (**D**), TMOD (**E**), and the N terminus of β-spectrin (**F**) clusters. Insets: two-dimensional autocorrelation for the magenta and orange boxes in (**A**), (**B**), and (**C**), showing quasi-rectangular lattices for TMOD and the N terminus of β-spectrin, but not for CD47. (**G**–**I**) Voronoï diagram analysis of CD47 (**G**), TMOD (**H**) and the N terminus of β-spectrin (**I**). By analyzing the Voronoï polygonal area and fitting the distribution histogram with gamma function, the x-axis coordinates corresponding to the peaks of the fitted curves (x_{peak}) was obtained. The values of x_{peak} reflect the spatial distribution characteristics of the points. The more uniformly the points distributed, the closer the value of x_{peak} is to 1. x_{peak} for CD47 is 0.8, while that of both TMOD and the N terminus of β-spectrin are 0.86, indicating that cytoskeletal proteins distributed more uniformly than CD47. (**J**) The number of STORM-resolved clusters of CD47, TMOD, and N terminus of β-spectrin per unit area of the erythrocyte membrane. Each data point corresponds to the result from one cell. (**K**) Simulation of random distributed disks (15 nm radius) with the same molecular densities of CD47 per unit area and the corresponding Voronoï diagram. (**L**) Distributions of nearest-neighbor distance of the simulated disks. (**M**) The Voronoï polygon results of random-distributed disks. The coordinates of the x-axis corresponding to the peaks of the Voronoï polygonal area distribution (~0.79) is close to that of CD47 (~0.8).

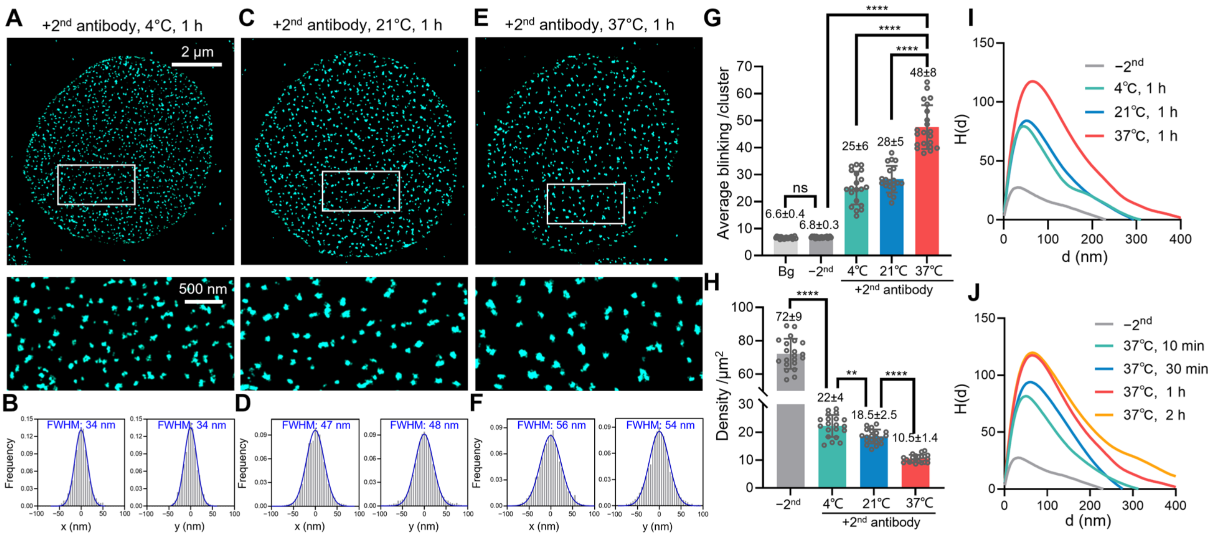

Supplementary Figure S3 Temperature dependence of the secondary antibody-induced aggregation of CD47 on the erythrocyte membrane. (**A**–**F**) Representative STORM images (**A**, **C**, and **E**) and resultant distributions of single molecule localizations of overlaid clusters (**B**, **D**, and **F**) for samples after the application of the secondary antibody at 4°C, 21°C, 37°C for 1 h, respectively. (**G**) Average counts of single-molecule blinking events detected for each cluster labeled by the CD47 primary antibody, for erythrocytes not crosslinked by the 2nd antibody (-2nd) and crosslinked at different temperatures for 1 h, in comparison to that for isolated single antibodies found on the coverslip (Bg). Each data point corresponds to the average count from clusters in one cell. (**H**) Distribution of the number of STORM-resolved CD47 clusters per unit area of the erythrocyte membrane, for samples without (-2nd) or with the secondary antibody treatment at different temperatures for 1 h. Each data point corresponds to the result from one cell. (**I**–**J**) Ripley's K analysis for erythrocytes treated with the secondary antibody at different temperatures for 1 h (**I**) or with the secondary antibody treatment at 37°C for different durations (**J**), indicating gradually increased cluster sizes and spacings after the treatments.

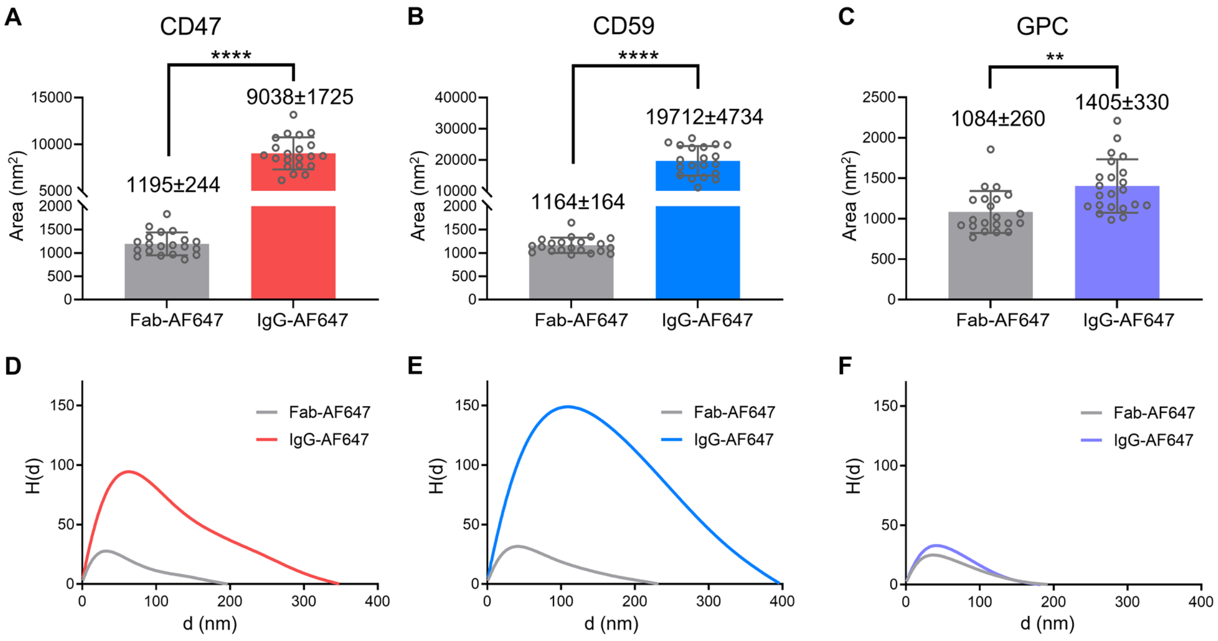

Supplementary Figure S4 Cluster area and Ripley's K analysis for secondary antibody-induced aggregation of CD47, CD59, and GPC in erythrocytes. (**A**–**C**) Statistics of the average area of clusters in STROM images of erythrocytes incubated with untagged anti-CD47 (**A**), anti-CD59 (**B**), and anti-GPC (**C**) primary antibodies, and then labeled with a dye-tagged Fab (left) or a dye-tagged full IgG secondary antibody (right). Each data point corresponds to the result from one cell. **(D**–**F)** Ripley's K analysis for STROM images of erythrocytes incubated with untagged anti-CD47 (**D**), anti-CD59 (**E**), and anti-GPC (**F**) primary antibodies, and then labeled with a dye-tagged Fab (gray curves) or a dye-tagged full IgG secondary antibody (red, blue and violet curves).

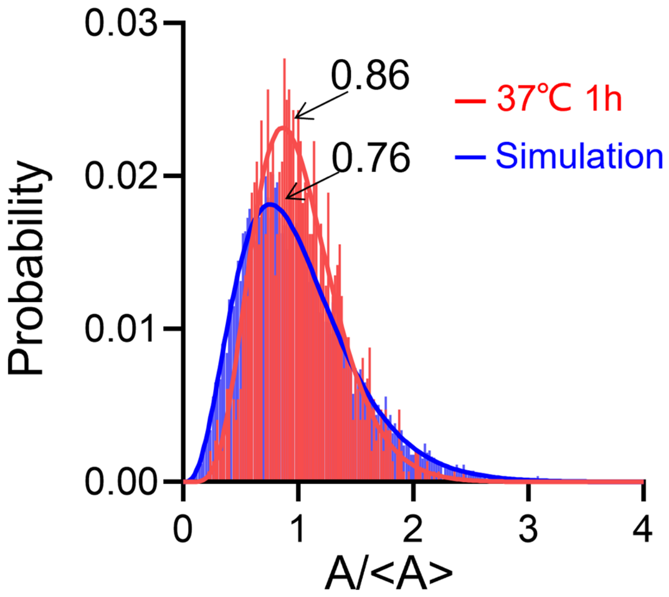

Supplementary Figure S5 Voronoï diagram analysis of secondary antibody cross-linking CD47 clusters in erythrocytes. The coordinates of the x-axis corresponding to the peaks of the Voronoï polygonal area distribution is 0.86 for secondary antibody cross-linking CD47 clusters and 0.76 for simulated clusters with random distribution, revealing that secondary antibody cross-linking CD47 distributed uniformly across the membrane, similar with that of cytoskeleton proteins with triangular lattice pattern (0.86 for both TMOD and N terminus of β-spectrin). This supporting the possibility that the clustering of CD47 might be associated with cytoskeleton.

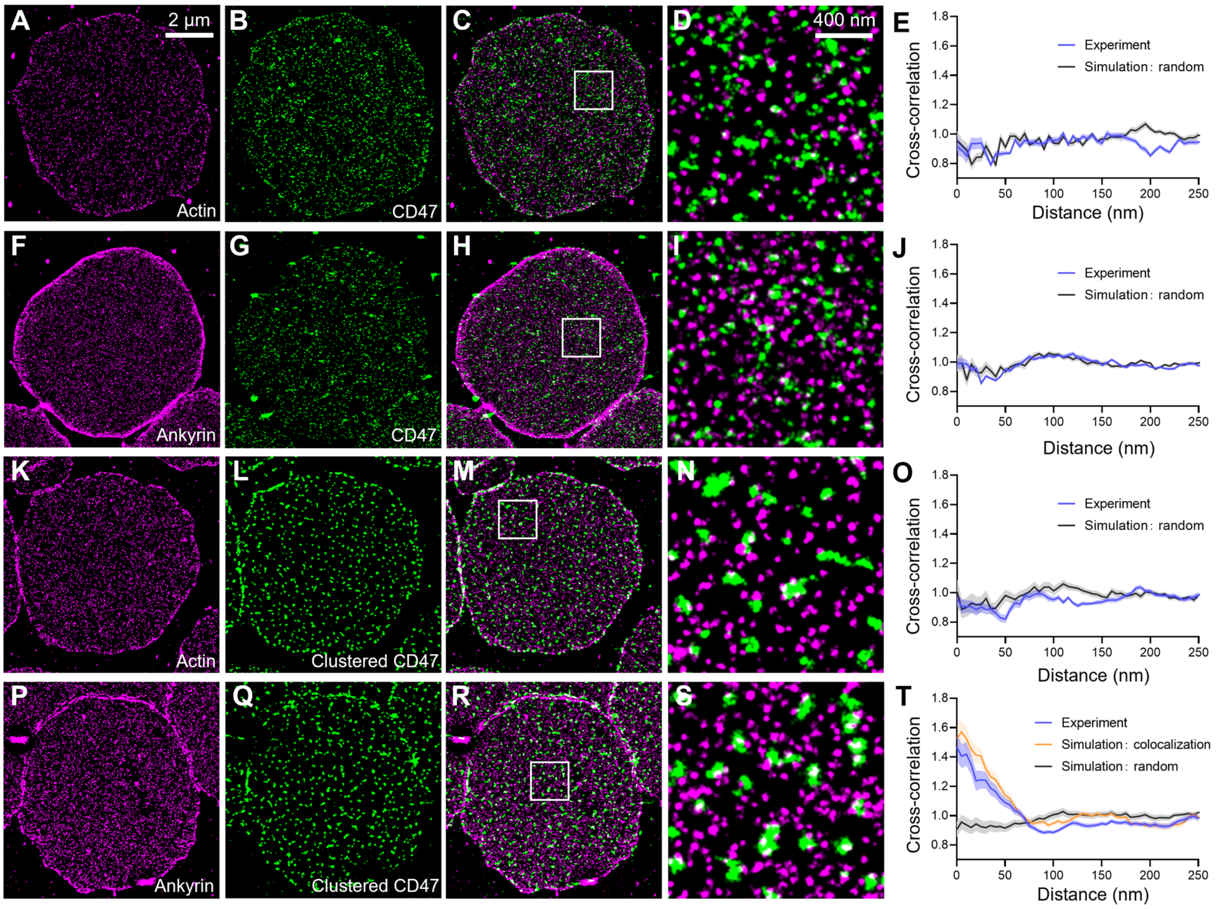

Supplementary Figure S6 Colocalization analysis of native and clustered CD47 caused by 2nd antibody and membrane cytoskeleton-associated proteins based on two-color STORM. (**A**–**C**) Representative STORM images of actin (**A**; magenta), CD47 (**B**; green), and overlaid image (**C**). (**D**) Zoom-in of the white box region in (**C**). (**E**) Cross-correlations between the two channels at different intermolecular distances calculated using experimental (blue) and simulated (black) data. Error bars indicate the SD between ten sets of simulated data. (**F**–**H**) Representative STORM images of ankyrin (**F**; magenta), CD47 (**G**; green), and overlaid image (**H**). (**I**) Zoom-in of the white box region in (**H**). (**J**) Cross-correlations between the two channels at different intermolecular distances calculated using experimental (blue) and simulated (black) data. Error bars indicate the SD between ten sets of simulated data. (**K**–**M**) STORM images of actin (**K**; magenta), clustered CD47 induced by 2nd antibody (**L**; green), and overlaid image (**M**). (**N**) Zoom-in of the white box region in (**M**). (**O**) Cross-correlations between the two channels at different intermolecular distances calculated using the experimental data (blue) and simulated data for random distributions (black). Error bars indicate the SD between ten sets of simulated data. (**P**–**R**) STORM images of ankyrin (**P**; magenta), clustered CD47 induced by 2nd antibody (**Q**; green), and the overlaid image (**R**). (**S**) Zoom-in of the white box region in (**R**). (**T**) Cross-correlations between the two channels at different intermolecular distances were calculated using the experimental (blue) data and simulated data for random distributions (black) or colocalized distributions at the measured cluster size and density (orange). Error bars indicate the SD between ten sets of simulated data.

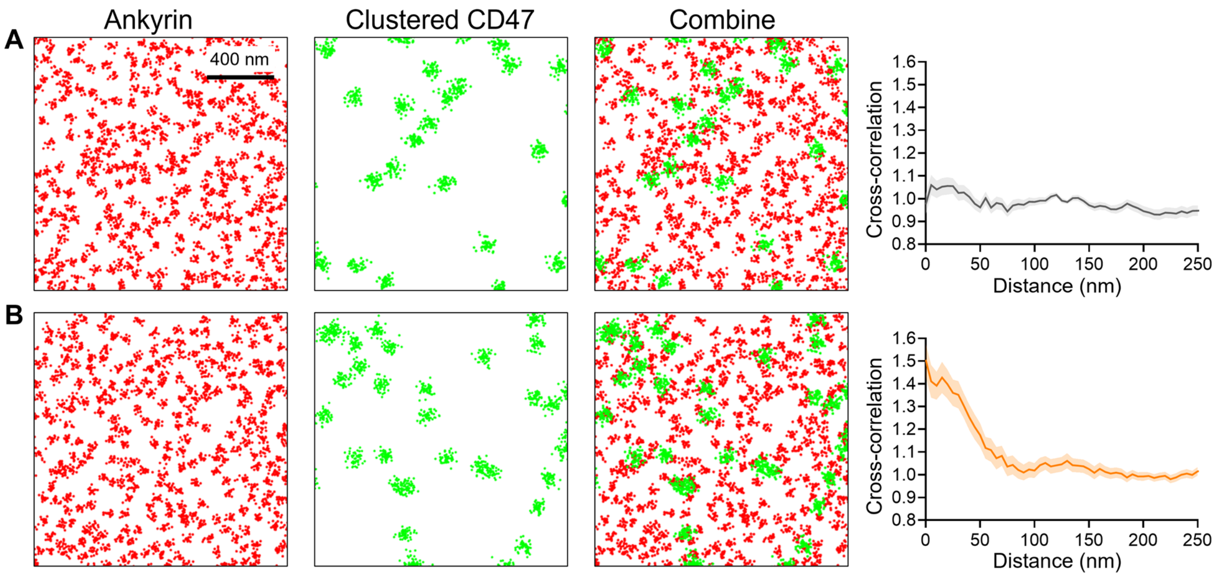

Supplementary Figure S7 Simulation of two-color STORM-based co-localization analysis. Two types of clusters namely ankyrin (red) and clustered CD47 (green) were created for simulation. The cluster density and the blinking number of clusters were set the same as the experimental results. The clusters of the two channels were arranged to be randomly distributed (**A**) or fully co-located (**B**). (**A**) Cross-correction result of ankyrin and clustered CD47 when they are randomly distributed. The value of cross-correlation fluctuates around 1. (**B**) Cross-correction result of ankyrin and clustered CD47 when they are fully co-localized. The value of cross-correlation reaches 1.5 at x=0, gradually decreases and finally fluctuates around 1.

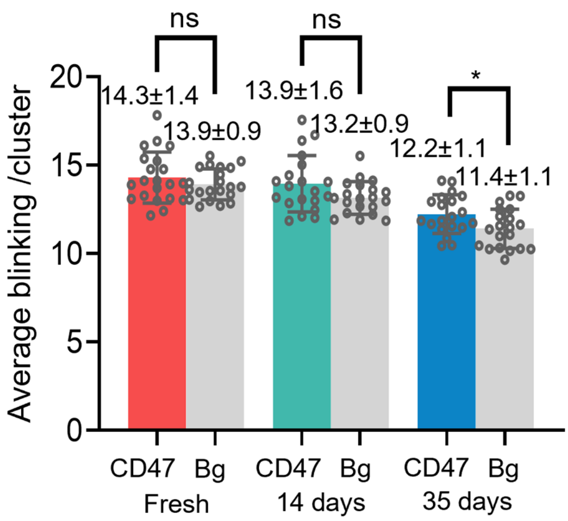

Supplementary Figure S8 CD47 maintains as monomers in the human erythrocytes during aging. The average counts of single-molecule blinking events detected for each cluster labeled by the CD47 primary antibody in fresh, 14-day storage, and 35-day storage human erythrocytes, compared to that for isolated single antibodies on the coverslip (Bg). Note that for these samples, a 405 nm photoactivation laser is applied during imaging to ensure the blinking of the fluorescence probe. Consequently, different blinking counts are observed for the Bg signals.

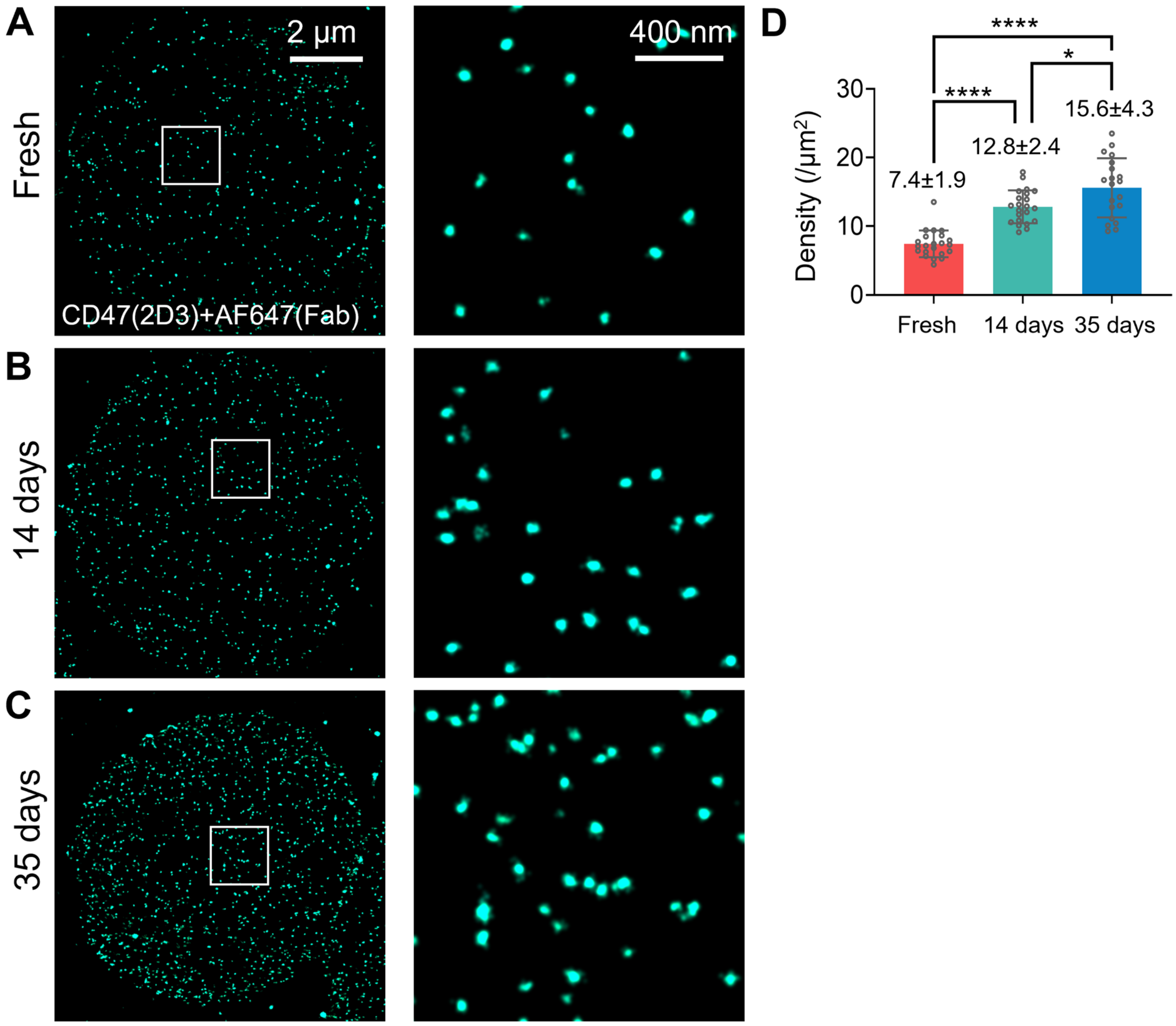

Supplementary Figure S9 The conformational change of CD47 during storage. (**A**–**C**) Representative STORM images of conformational changed CD47 molecules labeled by CD47 primary antibody (2D3 clone) in fresh (**A**), 14-day (**B**), and 35-day (**C**) storage erythrocytes and zoom-in of the white box region. (**D**) Statistical density of conformational changed CD47 in fresh, 14-day, and 35-day storage erythrocytes.

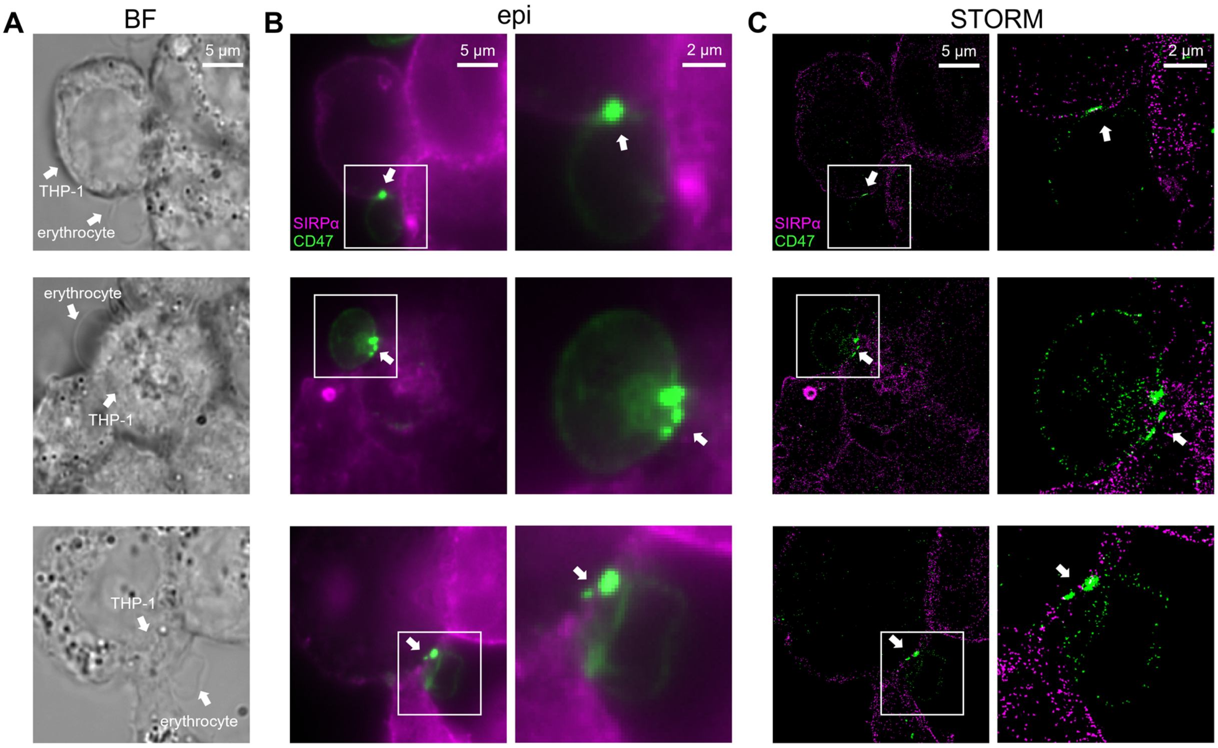

Supplementary Figure S10 Two-color STORM of CD47 and SIRPα in co-culture system. Representative BF(**A**), epi(**B**) and STORM(**C**) images of CD47 clusters on cell-cell conjugate sites between erythrocytes and THP-1-derived macrophages.

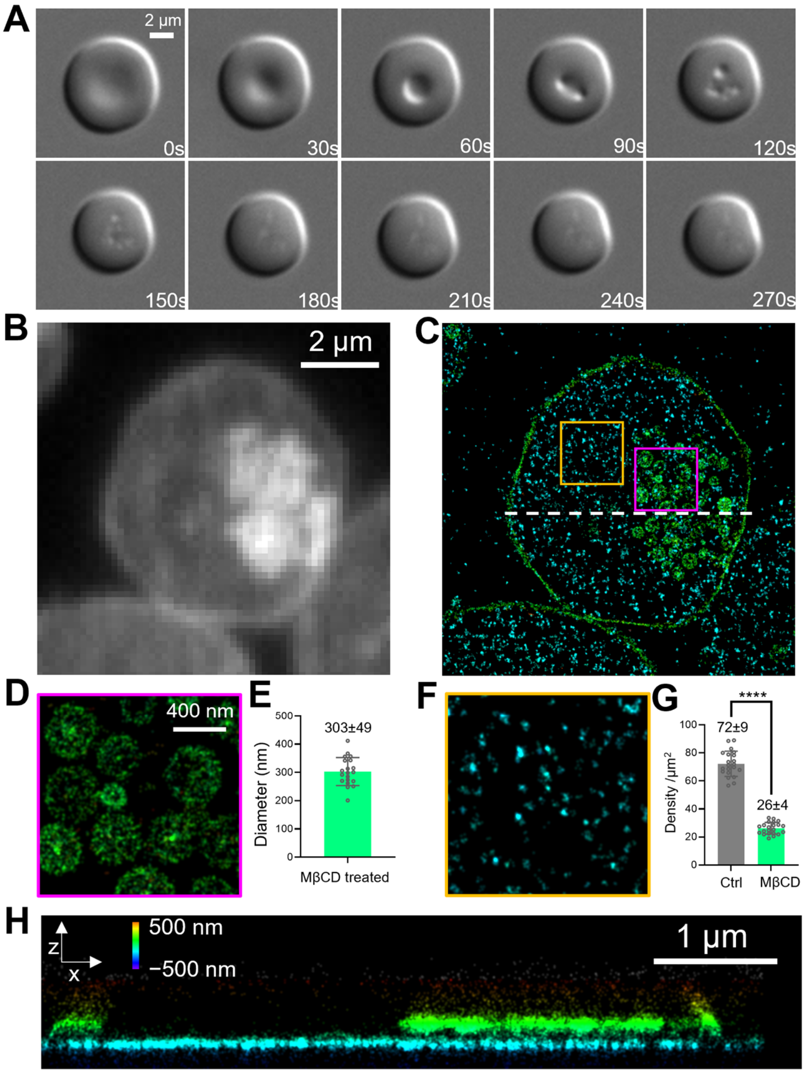

Supplementary Figure S11 The effect of methyl-β-cyclodextrin on CD47 organization in erythrocytes. (**A**) Time-lapse photography of fresh erythrocytes treated with 5 mM methyl-methyl-β-cyclodextrin. The biconcave shape of the erythrocytes gradually disappeared within 3 min. (**B**–**C**) Conventional fluorescence image (**B**) and STORM image (**C**) of CD47 in methyl-β-cyclodextrin-treated erythrocytes labeled with fluorescent primary antibody. (**D**) A magnified image of the magenta box region in (**C**) showing microvesicles caused by methyl-β-cyclodextrin. (**E**) Statistics of the diameters for microvesicles. (**F**) A magnified image of the orange box region in (**C**) showing the remaining CD47 on the methyl-β-cyclodextrin-treated erythrocyte membrane. (**G**) Statistics of the density of CD47 on the membrane of erythrocytes treated with and without methyl-β-cyclodextrin. (**H**) Virtual cross-section of the STORM image of CD47 in the *xz* plane along the white dashed line in (**C**). Microvesicles are located inside the erythrocytes.

Supplemental Materials and methods

Reagents

Methyl-β-cyclodextrin (C4555); 12-O-Tetradecanoylphorbol 13-acetate (PMA, P8139) were from Sigma-Aldrich.

Primary antibodies: Anti-SIRPα, rabbit monoclonal (ab191419), Anti-CD47 (2D3), mouse monoclonal (14-0478-82, Invitrogen).

Secondary antibody Fab fragments: Alexa Fluor 647 AffiniPure Fab fragment goat anti-rabbit IgG (H+L) (111-607-003, Jackson ImmunoResearch).

Sample Preparation of THP-1 & erythrocyte co-culture experiments

For THP-1 cell lines: suspended THP-1 cells were cultured in RPMI 1640 (10% FBS) medium. To obtain differentiated THP-1 cells, 100 ng/ml PMA was added to the medium and incubated for 24 h at 37 ℃.

For erythrocytes: fresh human fingertip blood was diluted in phosphate-buffered saline (PBS) containing 10 mM glucose and 10 mg/ml BSA (PBS-GB), centrifuged twice at 700× g, and resuspended in PBS-GB to obtain an erythrocyte suspension at ~5×10^5 cells/ml. The erythrocyte suspension was incubated with the primary antibodies (anti-CD47-CF583R) for 1 h at room temperature. The sample were centrifuged twice to remove the residual dye-tagged primary antibodies and resuspended in 500 μL of RPMI 1640 (10% FBS).

Labeled erythrocytes suspended in 500 μl of RPMI 1640 medium were added to differentiated THP-1 cells and incubated at 37°C for 1 h, followed by 4% PFA fixation for 20 min. Then, the samples were blocked in a blocking buffer (3% w/v BSA in PBS) for 20 min. Afterward, the cells were incubated with the primary antibody (Anti-SIRPα) in blocking buffer for 1 h. Next, the cells were washed with washing buffer (0.2% w/v BSA in PBS) three times and incubated with the secondary antibody Fab fragments for 1 h. After that, the samples were washed three times with washing buffer and then mounted for imaging.

Voronoï diagram analysis

The Voronoï diagram is constructed using MATLAB based on the center of mass of the membrane protein clusters obtained from clustering analysis of STORM images. The area of each Voronoï polygon is found and divided by its average area ($A/\langle A\rangle$), and then a histogram is made based on the normalized values. The obtained histogram satisfies the gamma distribution (Zaninetti, 1992; Ferenc and Néda, 2007). For randomly distributed ideal points, the maximum value of the gamma distribution corresponds to an x-axis coordinate of 0.72. When the points tend to be uniformly distributed, the corresponding x-axis coordinates tend to be close to 1.

Reference

Ferenc, J.-S., and Néda, Z. (2007). On the size distribution of Poisson Voronoi cells. Phys. Stat. Mech. Its Appl. *385*, 518–526.

Zaninetti, L. (1992). The Voronoi tessellation generated from different distributions of seeds. Phys. Lett. A *165*, 143–147.
